# A Low-Phase-Noise InGaAs/GaAs BiHEMT Balanced Colpitts VCO with Deep-Learning-Optimized On-Chip Inductor for Satellite Communication Applications

**DOI:** 10.3390/mi17101148

**Published:** 2026-09-30

**Authors:** Maolin Zhou, Xinlin Xia, Haiyang Zhu, Yanjie Wang

**Affiliations:** School of Microelectronics, South China University of Technology, Guangzhou 511442, China; 202110190882@mail.scut.edu.cn (M.Z.);

**Keywords:** balanced Colpitts VCO, deep learning, GaAs BiHEMT, low phase noise, noise-shifting

## Abstract

In this communication, a balanced Colpitts VCO with low phase noise and a wide tuning range is presented, which was implemented using a 2 μm GaAs BiHEMT process. To improve phase noise, a noise-shifting technique introduced by the cross-coupled pair was applied. A deep-learning-assisted genetic algorithm was employed to optimize the quality factor of an on-chip inductor, further improving the phase noise. Additionally, three-bit switches were utilized to expand the tuning range of this VCO. Finally, a VCO prototype was implemented and fabricated for verification. The measurement results demonstrate that the proposed VCO can achieve a wide tuning range of 33% (2.56–3.57 GHz) and an excellent phase noise of −136.25 dBc/Hz at a 1 MHz offset, validating the efficiency of the adopted circuit techniques and the machine learning-assisted optimization strategy for low-phase-noise design. Consequently, this VCO proves to be a highly competitive candidate for signal sources in *S*-band satellite communication systems.

## 1. Introduction

High-performance voltage-controlled oscillators (VCOs) with low power dissipation, wide frequency-tuning ranges, and low phase noise have attracted extensive attention in recent years. To date, numerous VCOs have been designed using various processes [1,2,3,4,5,6]. Among the reported VCOs, those based on Si-bulk technologies (CMOS/BiCMOS) have always been popular due to their design flexibility and low power consumption [1,2,3]. In [1], a VCO implemented in a CMOS process achieved a phase noise of −128.5 dBc/Hz at 1 MHz with a figure of merit (FOM) of −184.5 dB by employing a novel 8-shaped inductor. In [2], a Class-C VCO was designed and verified in a BiCMOS process. Its power consumption was reduced by the dynamic body-bias technique, resulting in a high FOM of −188.5 dBc.

Most Si-bulk-based VCOs have a high FOM, but their less-than-satisfactory phase noise, mainly caused by relatively high substrate loss and low-frequency flicker noise, may limit the application of silicon VCOs in scenarios that require extremely low phase noise. Compound-semiconductor VCOs have been widely investigated for low-phase-noise applications [4,5,6]. Their performance depends not only on the technology behind the device used but also on the feedback structure and resonator losses. Colpitts VCOs can offer favorable phase-noise performance, but their relatively stringent startup requirements often necessitate a high-Q tank inductor and a higher bias current, resulting in a tradeoff between reliable startup and power consumption. In contrast, conventional cross-coupled VCOs feature a simple structure and relatively easy startup, although their phase-noise performance can be less favorable under comparable operating conditions. A VCO implemented with GaAs HBT technology was reported in [4]. This design adopts a single-ended Colpitts architecture and achieves an ultra-low phase noise of −135.4 dBc/Hz at 1 MHz offset, at the cost of high DC power consumption amounting to 300 mW. In [5], a VCO was also realized in a GaAs BiHEMT process. This design adopts a differential architecture based on common-base/common-emitter configurations. The HBT is used as the oscillation core to achieve low phase noise, while the HEMT is adopted as the varactor for wide frequency tuning. Benefiting from this architecture, power consumption is reduced compared with the previously reported GaAs HBT VCOs. A GaN-on-SiC Hartley oscillator demonstrated potential for low phase noise and high output power [7]; however, its fixed-frequency implementation does not address the additional loading and performance variation associated with a wide tuning range. Although compound-semiconductor-based VCOs can achieve superior low-phase-noise performance, they generally exhibit higher power consumption compared with their silicon-based counterparts. In conventional tunable oscillators, varactors and switched-capacitor arrays extend the frequency-tuning range but inevitably introduce additional resistive loss and bias-dependent parasitic effects, further degrading the resonator quality factor. These inherent trade-offs motivate the separate optimization of active-core operation and passive resonator performance. Therefore, it remains challenging and highly desirable to develop a compound-semiconductor VCO that can simultaneously achieve excellent phase noise and a satisfactory FOM.

Traditionally, high-performance analog and RF circuit design heavily relies on iterative simulation tuning and empirical designer experience. Such manual optimization procedures are extremely time-consuming and struggle to yield globally optimal solutions, especially when balancing complicated trade-offs among multiple conflicting performance metrics. For VCO designs, the on-chip tank inductor dominates such design trade-offs since its geometric parameters simultaneously determine the inductance, quality factor, self-resonant frequency (SRF), and chip area. Adjusting the conductor width, turn spacing, or outer dimension inevitably alters multiple electrical characteristics at once. Therefore, simply maximizing the quality factor is insufficient; the inductor must also satisfy stringent constraints on target inductance, adequate SRF, layout area, and process design rules. Repeated electromagnetic (EM) simulations for various geometric candidates further make this multi-constrained manual design process computationally expensive and inefficient. Recent AI- and machine learning-assisted methods address different aspects of circuit optimization. Hybrid GA/ML methods have been applied to automated LNA design [8], while deep-learning-based inverse design has been used to synthesize on-chip electromagnetic structures and power-amplifier matching networks [9]. Knowledge-infused approaches combining Bayesian optimization and reinforcement learning have also been investigated for analog device-parameter optimization [10]. AI-assisted automated design has additionally been reported for multi-stage GaN-on-SiC MMIC power amplifiers [11]. More general inverse design frameworks extend the search to arbitrarily shaped multi-port electromagnetic structures and their integration with active circuits [12]. These approaches differ in their design variables, model outputs, and search spaces. The present work focuses on a fixed two-turn inductor topology and a bounded set of geometrical variables, with explicit inductance, SRF, area, and manufacturability constraints. A forward DNN surrogate is combined with a GA to evaluate and search candidate geometries.

This communication presents a Colpitts VCO implemented using the GaAs BiHEMT process. A noise-shifting technique introduced by a cross-coupled pair is adopted to improve the quality factor (Q). A deep-learning algorithm is employed to optimize the design of an inductor, aiming to enhance its quality factor (Q) without a substantial area increase while maintaining appropriate values for both inductance (L) and self-resonant frequency (SRF). Consequently, better phase-noise performance can be achieved. Additionally, to expand the tuning range, a three-bit switch is implemented using E-PHEMT. The results obtained demonstrate that both the phase noise and the figure of merit (FOM)/figure of merit including tuning range (FOMT) of this VCO have been optimized, representing a significant improvement compared with previous work.

## 2. VCO Analysis and Design

**Figure 1:**
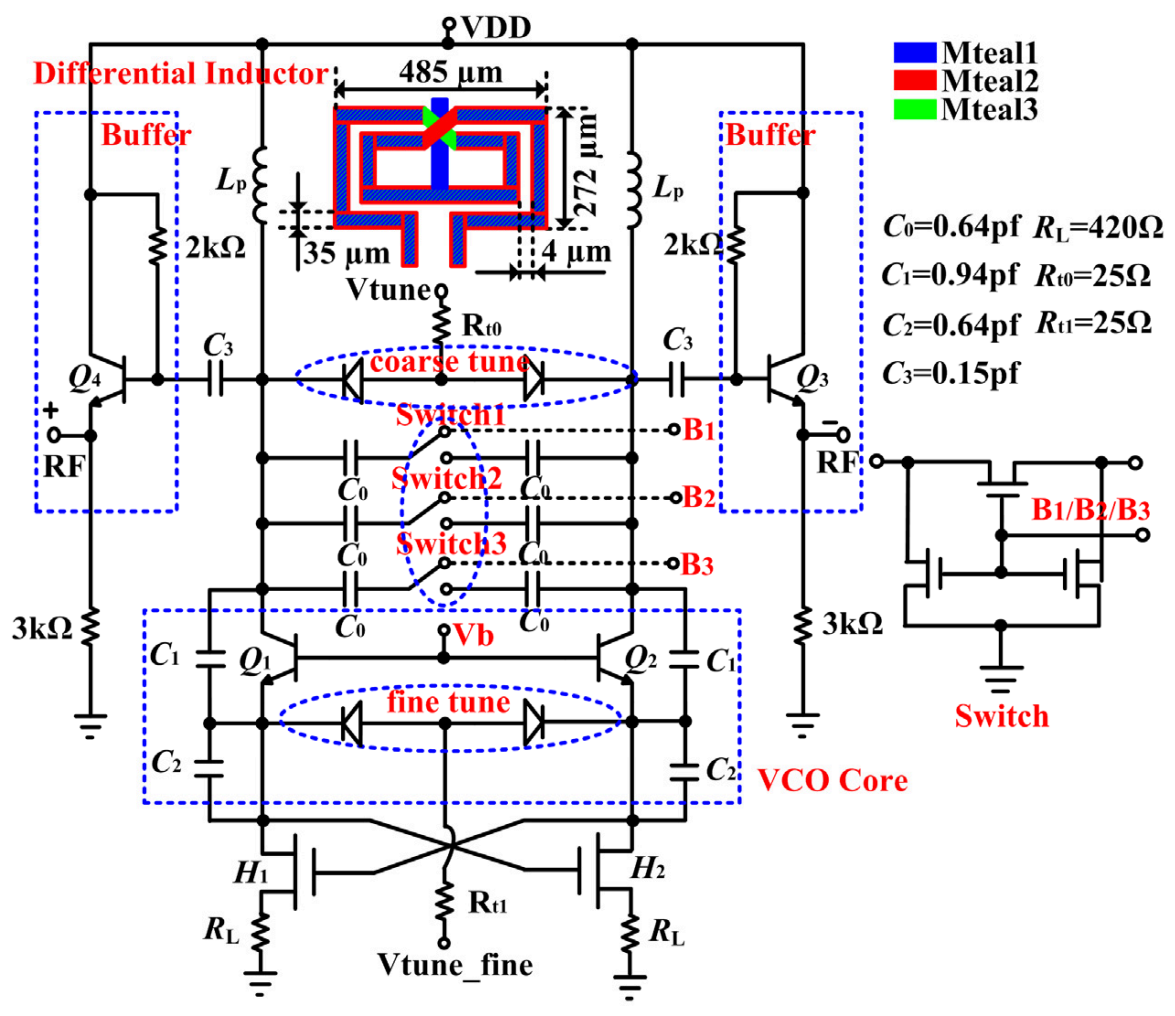
Schematic of the proposed balanced Colpitts VCO.

The topology of the presented VCO, shown in Figure 1, follows the noise-shifting balanced Colpitts architecture from our prior publication [13], while the tank inductor is re-optimized with the DNN-GA framework developed in this work. The active devices (Q_1_ and Q_2_) in the VCO core are hetero-junction bipolar transistors (HBTs) because, compared with high-electron-mobility transistors (HEMTs), they have high transconductance (*g*_m_) and low flicker noise. The resistors R_t0_ and R_t1_ are used to suppress the common-mode noise. Note that the thermal noise generated by R_t0_ and R_t1_ mainly acts as common-mode interference and is strongly suppressed by the balanced Colpitts topology, leading to negligible phase-noise degradation. Additionally, to achieve low phase noise and robust start-up conditions, a noise-shifting technique introduced by a cross-coupled E-PHEMT pair is adopted. The H_1_–H_2_ cross-coupled pair serves as an auxiliary regenerative and noise-shifting element rather than the core oscillation unit. It provides negative conductance to compensate resonator loss and raises the tank equivalent quality factor to facilitate oscillator startup. As oscillation amplitude grows, the transistors operate in alternating conduction mode, which improves bias-current utilization and enables target performance with reduced bias.

According to the Lesson formula [14], the phase-noise-generation mechanisms can be expressed as

(1) $$L\left(\Delta \omega \right)\propto \frac{F}{{Q^{2}}_{\mathrm{tank}}}\frac{{4K}_{B}{\mathrm{TR}}_{P}}{{A^{2}}_{\mathrm{tank}}}{\left(\frac{\omega_{0}}{2\Delta \omega }\right)}^{2}$$

where *F* is the noise factor, *K*_B_ is the Boltzmann constant, *A*_tank_ is the oscillation amplitude, *T* is the temperature, and *Q*_tank_ is the tank loaded quality factor. Clearly, the *Q*_tank_ is an essential parameter that dominates the phase noise. The presence of a load resistor *R*_L_ reduces the quality factor of a parallel *LC* resonator (the tank for the VCO) from its unloaded value *Q*_0_ to a loaded value *Q*_tank_, which is determined by the following expression:

(2) $$Q_{\mathrm{tank}}=Q_{0}/\left(1+\frac{R_{P}}{R_{L}}\right)$$

where R_P_ denotes the equivalent parallel resistance of the LC tank. Consequently, the loaded quality factor (Q_tank_) can generally be improved through two principal approaches:

(1). Enhancing intrinsic tank quality.

This can be achieved by increasing the unloaded quality factor (Q_0_) of the LC resonator itself.

(2). Raising the load impedance.

This can be achieved by increasing the load resistance (R_L_) to minimize its damping effect on the resonant tank.

As a rule of thumb, the unloaded Q_0_ of the VCO’s resonator in this frequency band is dominated by the differential inductor. Consequently, inductor design is paramount to the VCO’s performance.

**Figure 2:**
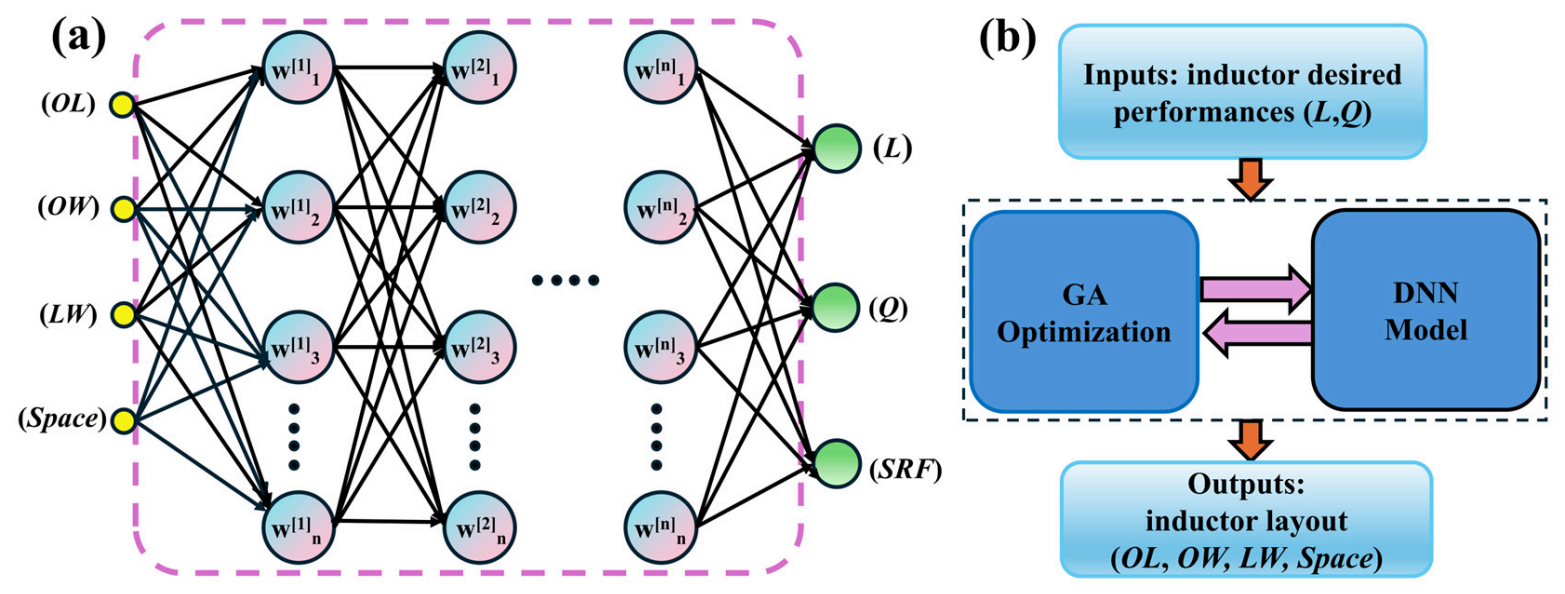
(**a**) DNN architecture: Inputs are four geometric parameters of spiral inductor (OL, OW, LW, Space); outputs are predicted electrical performance: inductance L, quality-factor Q and self-resonant frequency SRF. (**b**) Workflow of DNN-assisted GA inverse design for on-chip inductor: given target performance specifications, GA searches geometric parameters under DRC and performance constraints and outputs final inductor layout dimensions.

Designing a high-quality inductor is an intricate and time-consuming process, as critical parameters such as quality factor (Q), inductance (L), and self-resonant frequency (SRF)—all of which are vital for VCO performance—are determined by its physical dimensions, including metal width, turn-to-turn spacing, and coil diameter. These performance metrics are mutually conflicting: improving one parameter often comes at the cost of degrading another, making it difficult for manual iteration to find a geometry that satisfies all requirements simultaneously. In this work, a deep neural network (DNN) is proposed as a surrogate model to enable efficient prediction and optimization of the two-turn inductor’s performance metrics. The surrogate DNN architecture is illustrated in Figure 2a. The surrogate model is implemented as a multi-layer perceptron (MLP) that approximates the forward mapping f: (OL, OW, LW, Space) → (L, Q, SRF), where OL, OW, LW, and Space denote the inductor’s out length, out width, and line width and the spacing between adjacent turns, respectively, while L, Q, and SRF represent the inductance, quality factor, and self-resonant frequency.

The MLP architecture consists of an input layer with four nodes, eight hidden layers with 256 ReLU-activated neurons each, and an output layer with three nodes. The model is trained using the mean squared error (MSE) loss function and the Adam optimizer, with a learning rate of 2 × 10^−4^. Dropout with a probability of 0.2 is applied after each fully connected layer to prevent overfitting. The network is trained for 200 epochs on a dataset of 30,000 ADS-simulated samples, and the entire training process takes approximately 8 h on an NVIDIA RTX 3090 GPU.

Manual inductor design heavily relies on iterative electromagnetic (EM) simulation and empirical tuning. These four physical geometry parameters are strongly associated with conflicting requirements: increasing the metal line width LW can improve Q by reducing series resistance, yet it simultaneously raises parasitic capacitance and degrades the self-resonant frequency (SRF). Reducing turn spacing helps us obtain higher inductance L, but it introduces extra inter-turn parasitic capacitance and suppresses SRF as well. It is non-trivial for human designers to find a geometry combination that simultaneously satisfies target inductance, high Q, a sufficiently high SRF, and DRC layout rules, which requires dozens of time-consuming EM simulation runs.

The trained DNN is integrated with a genetic algorithm (GA) to explore the optimal geometric parameters under practical design constraints, including DRC rules, target inductance, the minimum SRF requirement, and the fixed maximum inductor area. The optimization objectives are set to maximize the inductor quality factor Q, subject to multiple hard constraints:

(1) Target inductance must be close to 600 pH for the targeted *S*-band oscillation frequency;
(2) The self-resonant frequency (SRF) > 12 GHz, ensuring the SRF is sufficiently higher than the VCO operating frequency range (2.56–3.57 GHz);
(3) All geometry parameters must satisfy GaAs BiHEMT process DRC design-rule constraints;
(4) The overall inductor area cannot exceed the area of the manually designed counterpart to avoid chip-area penalties.

The GA is initialized with 10,000 feasible candidates that satisfy all DRC constraints. A population size of 1024 is maintained for 30 generations. Tournament-based parent selection (tournament size: 4), crossover (rate: 0.8), and mutation (with an initial rate of 0.2, which is gradually reduced) are applied in each generation, while the top 50 candidates are preserved through elitism. Benefiting from the fast inference of the DNN surrogate model instead of repeated time-costly electromagnetic simulations, the GA converges to feasible geometric parameters within approximately two minutes.

The complete dataset was randomly divided into training, validation, and test subsets in a ratio of 70%/15%/15%. The test subset was kept completely independent from model training and validation and used only for evaluating the generalization capability of the trained DNN. The quantitative prediction results on the held-out test set are summarized in Table 1.

**Figure 3:**
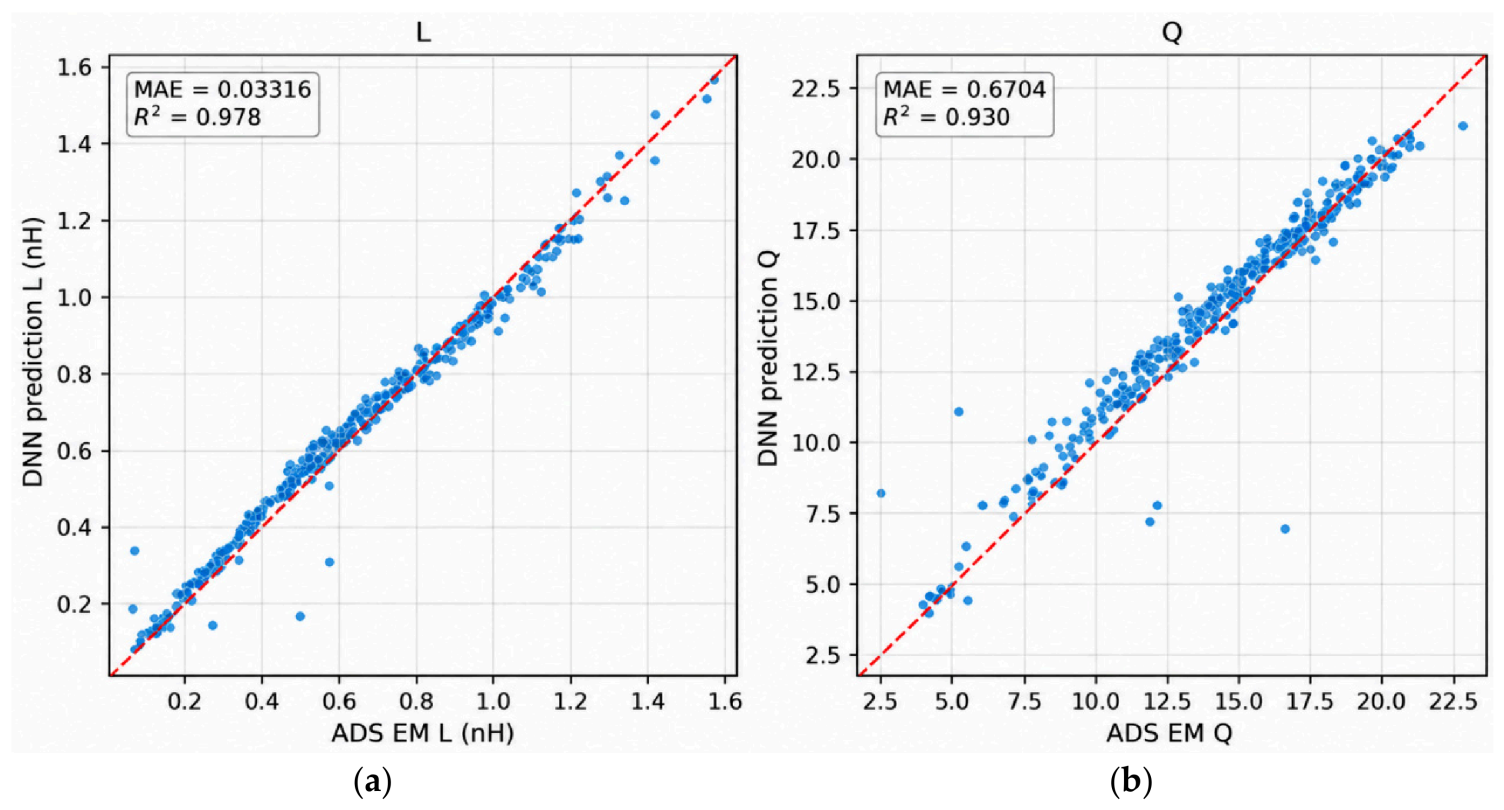
Parity plots of DNN predictions versus ADS EM simulation results on the held-out test set: (**a**) inductance (denoted as L) and (**b**) quality factor (denoted as Q).

The results indicate that the DNN surrogate model provides good prediction accuracy for all three target quantities. In particular, R2 values of 0.978, 0.930, and 0.909 were obtained for L, Q, and SRF, respectively. The corresponding parity plots for *L* and Q are shown in Figure 3, where the DNN predictions are closely distributed around the ideal *y* = *x* line.

To further evaluate the robustness of the surrogate model near the boundaries of the geometrical design space, the held-out test samples were classified into interior, edge, and corner regions. A sample was defined as an edge sample when at least one geometrical parameter was located within the lower or upper 10% of its allowable design range. A corner sample was defined as one for which at least two geometrical parameters simultaneously fell within their respective outer 10% ranges. The prediction errors for the three regions are summarized in Table 2.

These results show that the prediction errors near the design-space boundaries remain comparable to those in the interior region. No significant degradation can be observed for L or Q, while the SRF prediction error increases only moderately from 1.96% in the interior region to 2.96% in the corner region. This confirms that the surrogate model maintains satisfactory prediction accuracy near the geometrical boundaries, where the GA may tend to locate candidate optima.

**Figure 4:**
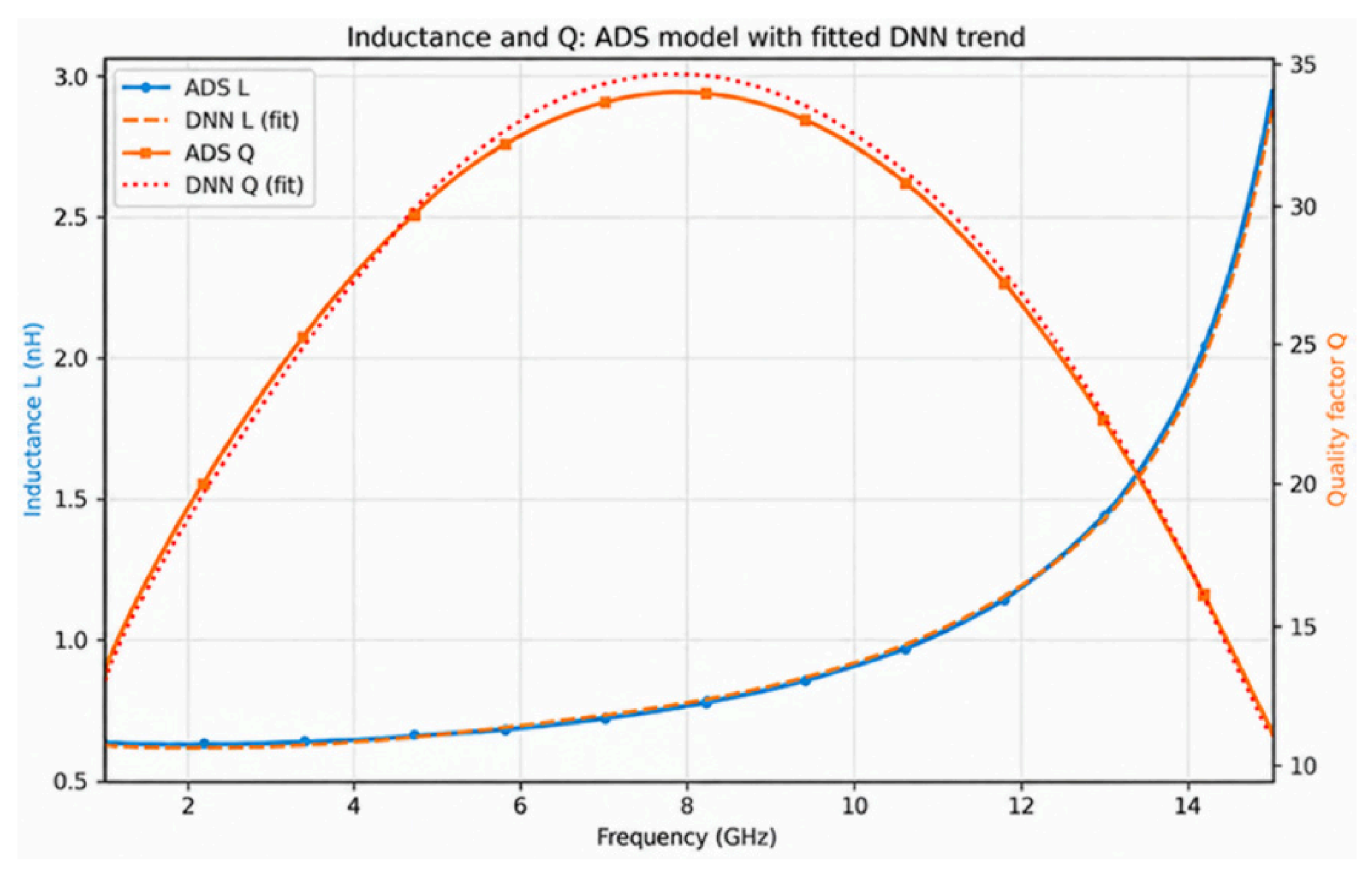
Comparison of the frequency-dependent inductance (L) and quality factor (Q) obtained from ADS EM simulation and the DNN surrogate model for a representative inductor design.

As an additional representative verification, the ADS EM results are compared with the DNN-predicted characteristics of the selected inductor design. Figure 4 presents the frequency-dependent inductance and quality factor. At 3.3 GHz, the ADS EM simulation yields an inductance of 630 pH and a quality factor of 25, showing close agreement with the corresponding DNN prediction. This comparison further demonstrates that the surrogate model can accurately reproduce the electrical characteristics of the inductor in the frequency region of interest.

**Figure 5:**
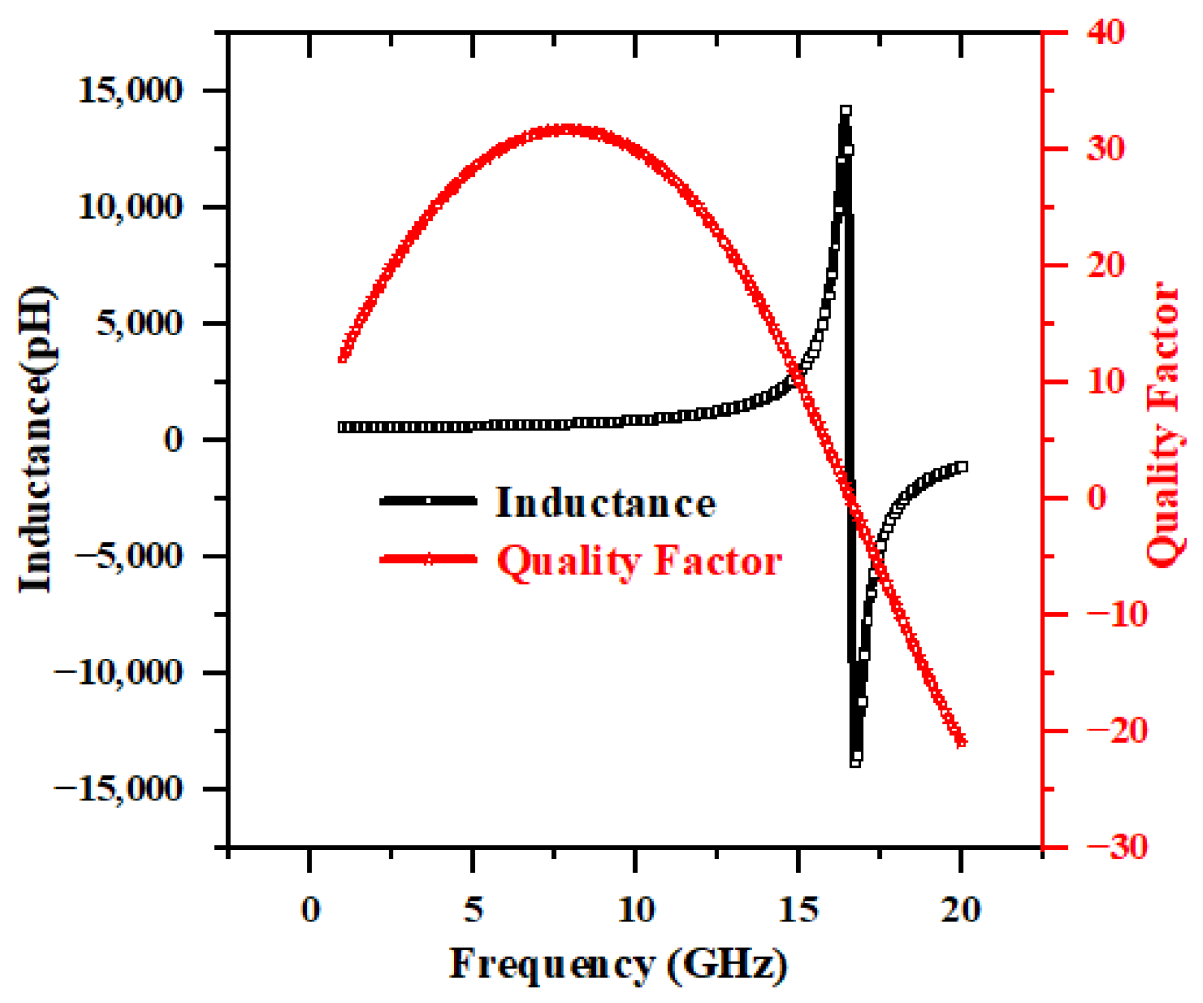
The EM-simulated frequency-dependent L and Q.

Figure 5 shows the inductance and Q factor of the differential inductor over the frequency range of 1–20 GHz based on a full-wave ADS EM simulation after GA layout generation.

In this study, the tank inductor was implemented as a two-turn structure, achieving a high quality factor of 25, an inductance of 630 pH, and a self-resonant frequency of 16 GHz. The enhanced unloaded quality factor Q_0_ elevates the loaded quality factor of the LC tank, which, together with the cross-coupled noise-shifting technique, contributes to the improved phase-noise performance of the proposed VCO. It is worth noting that the deep-learning workflow is only applied for passive tank-inductor geometry optimization; the active VCO core adopts conventional circuit design methods.

**Figure 6:**
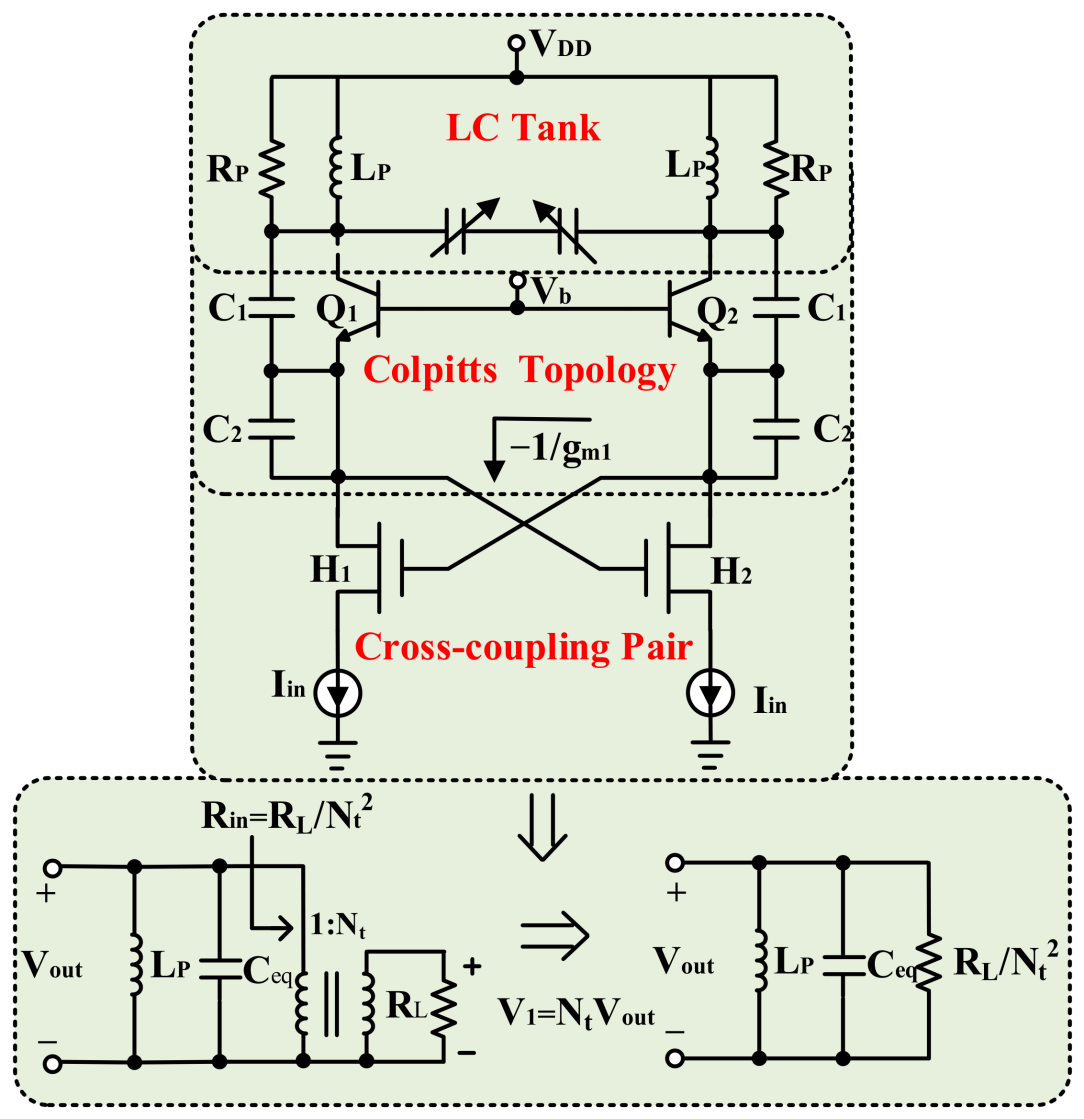
The equivalent transformer circuits of the proposed VCO.

In the practical design, the impedance observed when looking out from the emitters of Q_1_ and Q_2_ is not infinite. Therefore, the Q_tank_ would decrease due to the loading effect on the tank, which in turn deteriorates the phase noise. As indicated in Equation (2), the loaded Q_tank_ can be enhanced by increasing the load resistance R_L_ through mitigating the degrading effect of the load. However, in this Colpitts VCO topology (Figure 1), a higher *R*_L_ also reduces the transconductance (g_m_). This introduces a critical design trade-off, as an excessively large *R*_L_ may lead to a failure to oscillate due to the stringent startup condition of the Colpitts VCO. In this work, a cross-coupled pair (H_1_ and H_2_) is applied to improve the Q_tank_. The proposed VCO can be equivalent to a capacitive transformer, as shown in Figure 6. The resistor R_L_(−1/g_m1_) represents the impedance observed when looking out from the emitter of Q_1_ or Q_2_.

The transformation ratio (N_t_) from V_1_ to V_out_ is defined by Equation (2).

(3) $$N_{t}=C_{1}/\left(C_{1}+C_{2}+j\frac{g_{m1}}{\omega }\right)$$

where *g*_m1_ is the transconductance of H_1_ and H_2_.

The relationship between Q_tank_eff_ and Q_0_ can be defined by Equation (3)

(4) Qtank_eff=Q0/1+Nt2RPRL=Q0/1+|Nt|2RP−1gm1            =Q0/1−C12gm1RP(C1+C2)2+(gm1/ω)2≥Q0

where Q_0_ is the unloaded Q of the tank.

In contrast, after introducing the cross-coupled pair H_1_/H_2_, the active pair provides an effective negative conductance (−1/g_m1_) that partially compensates for the resonator loss. Meanwhile, the Colpitts capacitive network transforms this active impedance applied to the LC tank. Therefore, the increase in the effective tank Q_tank_eff_ in Equation (4) originates from active loss compensation rather than an increase in the intrinsic passive Q_0_ of the resonator. Consequently, lower phase noise can be achieved as per Equation (1).

The effective-Q enhancement in Equation (4) is conditional and requires the following constraint within the adopted reduced model:


(5) $$0<\frac{{C_{1}}^{2}g_{m1}}{{\left(C_{1}+C_{2}\right)}^{2}+({g_{m1}/\omega )}^{2}}<\frac{1}{R_{p}}$$

To clarify the phase-noise contributions of inductor optimization and the cross-coupled noise-shifting pair, we provide three sets of simulation results below: (a) a conventional Colpitts VCO with the manually designed inductor; (b) a conventional Colpitts VCO with the DNN-GA-optimized inductor; and (c) the complete proposed design incorporating both the optimized inductor and the cross-coupled noise-shifting pair.

**Figure 7:**
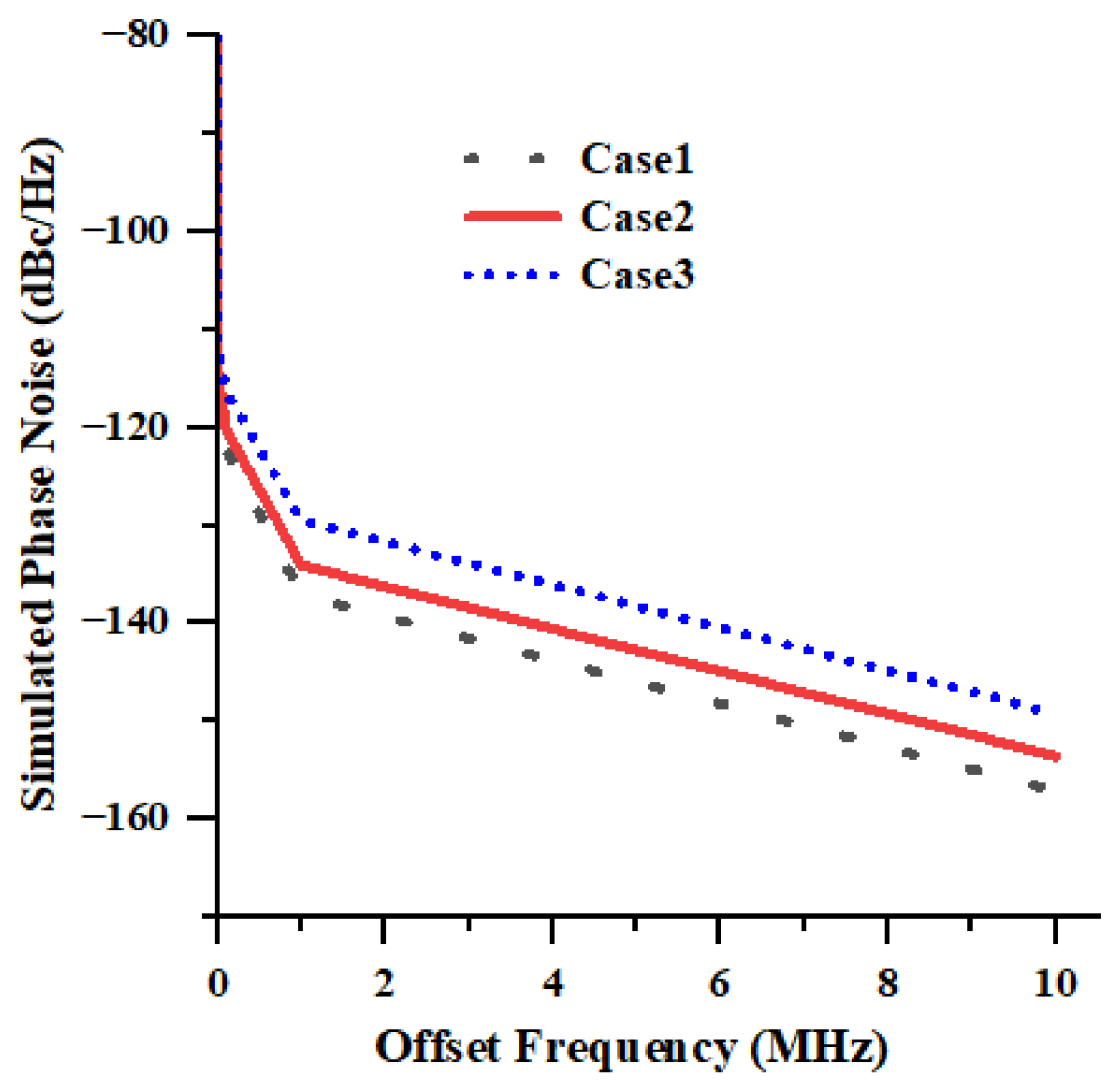
Simulated phase noise of three VCO cases: Case 1—the full proposed design; Case 2—a conventional Colpitts VCO with the DNN-GA-optimized inductor; and Case 3—a conventional Colpitts VCO.

As shown in Figure 7, at a 1 MHz offset, the simulated phase noise is −132.2 dBc/Hz, −135.6 dBc/Hz, and −137.1 dBc/Hz dBc/Hz, respectively. Replacing the manually designed inductor with the optimized inductor improves the phase noise by 3.4 dB. Introducing the cross-coupled noise-shifting pair while retaining the optimized inductor provides a further improvement of 1.5 dB.

## 3. Measurement Results and Discussion

**Figure 8:**
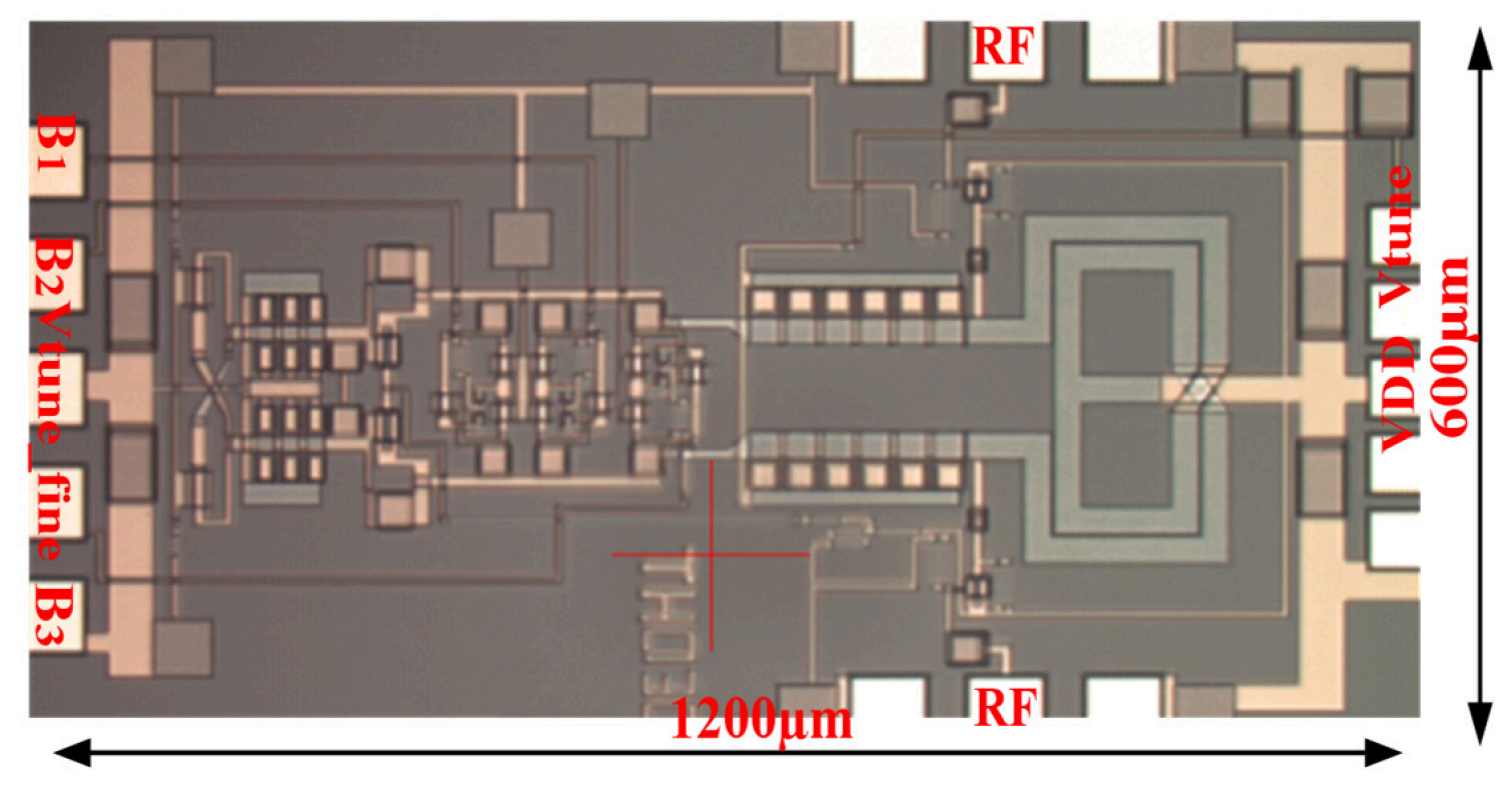
Microphotograph of the fabricated VCO.

**Figure 9:**
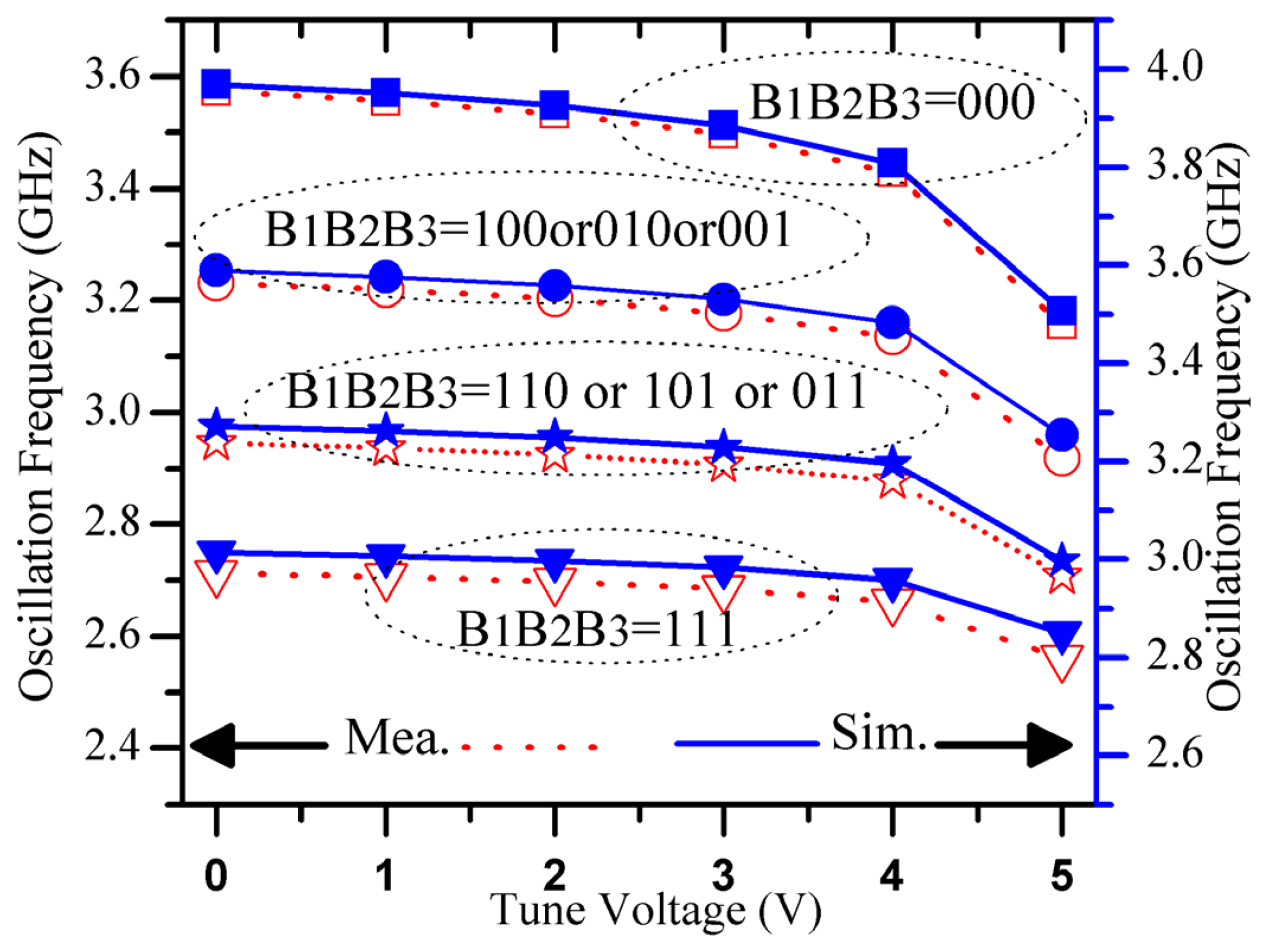
The measured and simulated oscillation frequency of the VCO (“0” means 0 V; “1” means 5 V).

**Figure 10:**
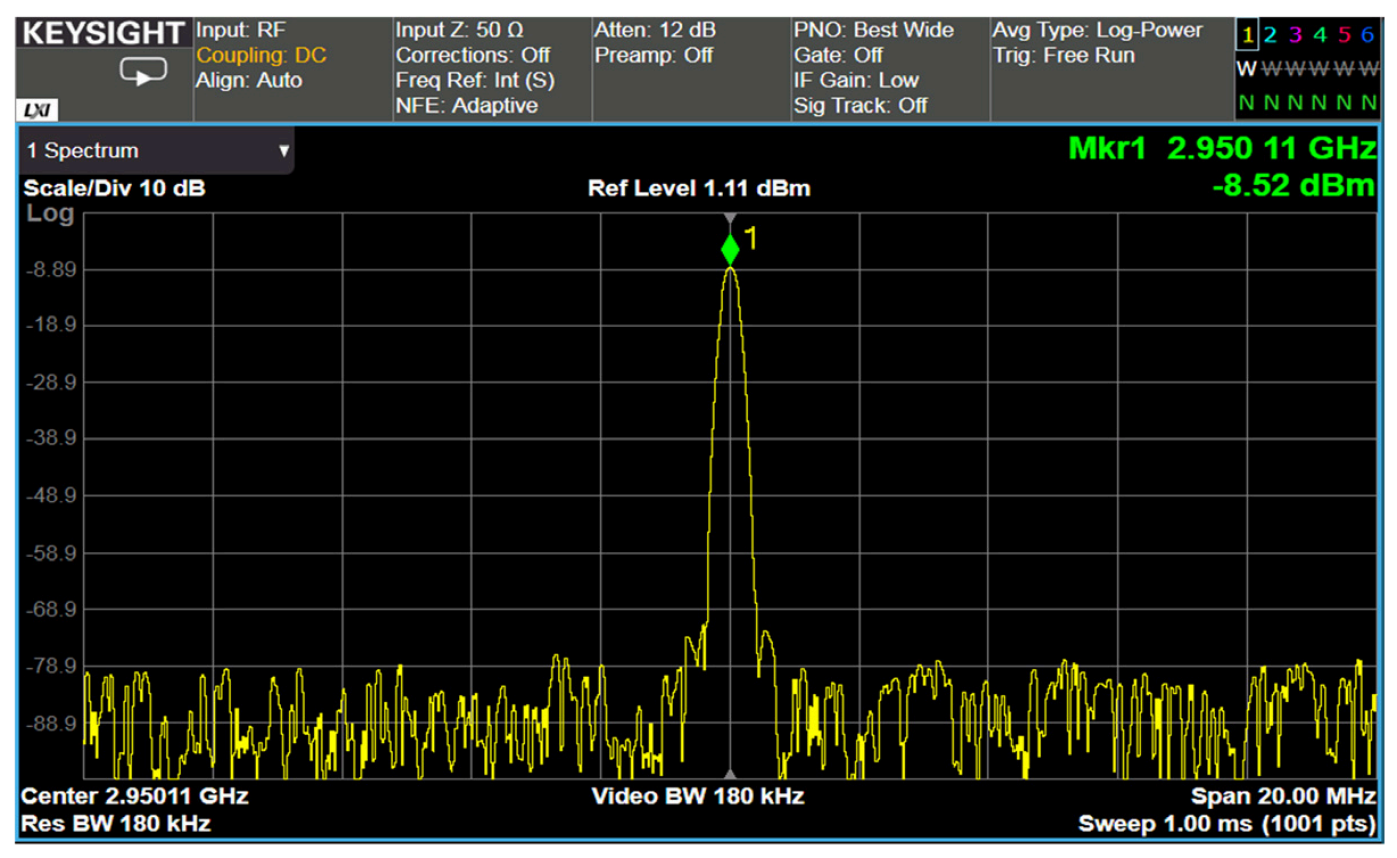
The measured frequency spectrum at 2.95 GHz.

**Figure 11:**
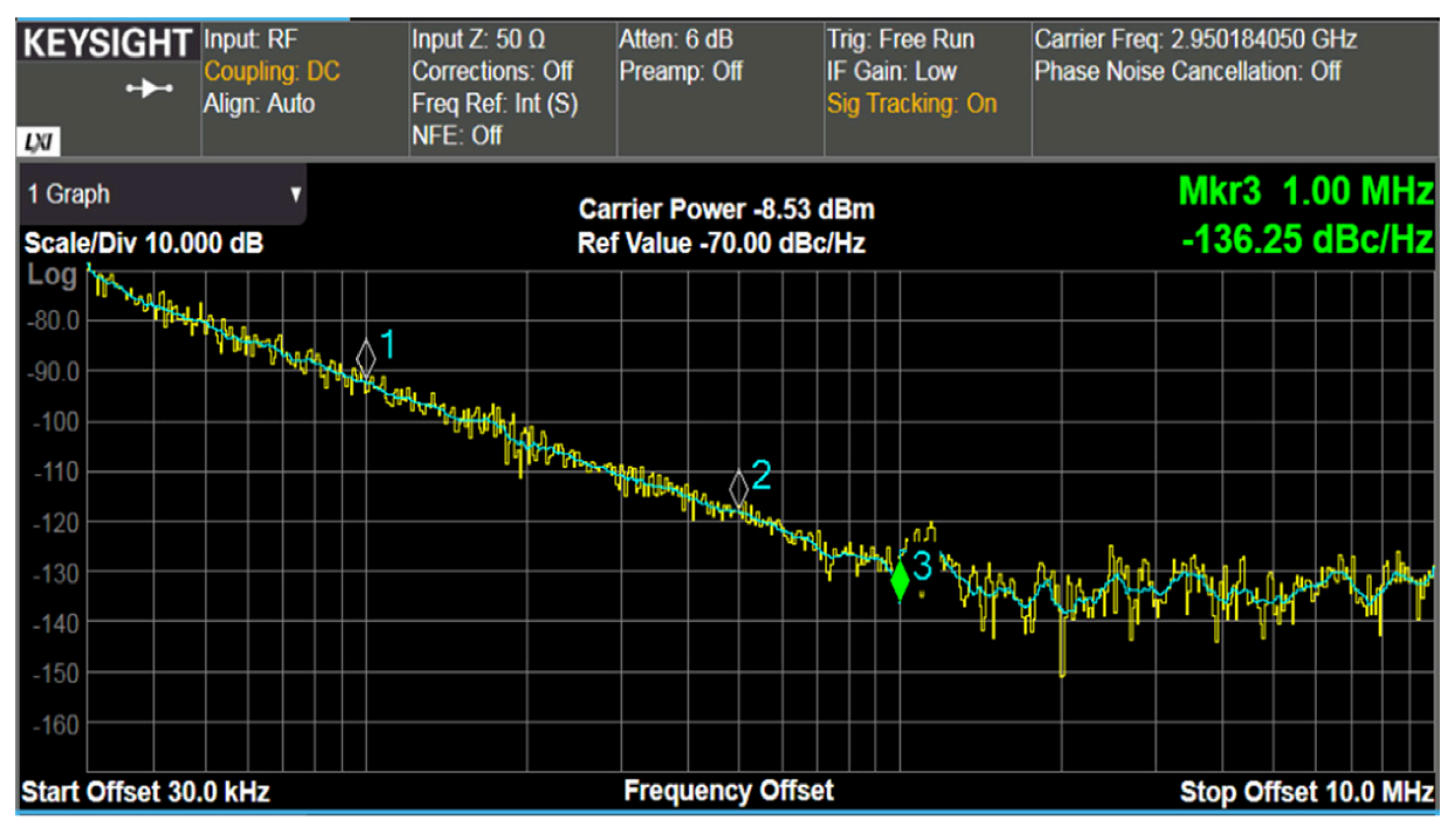
The measured phase noise at 2.95 GHz.

**Figure 12:**
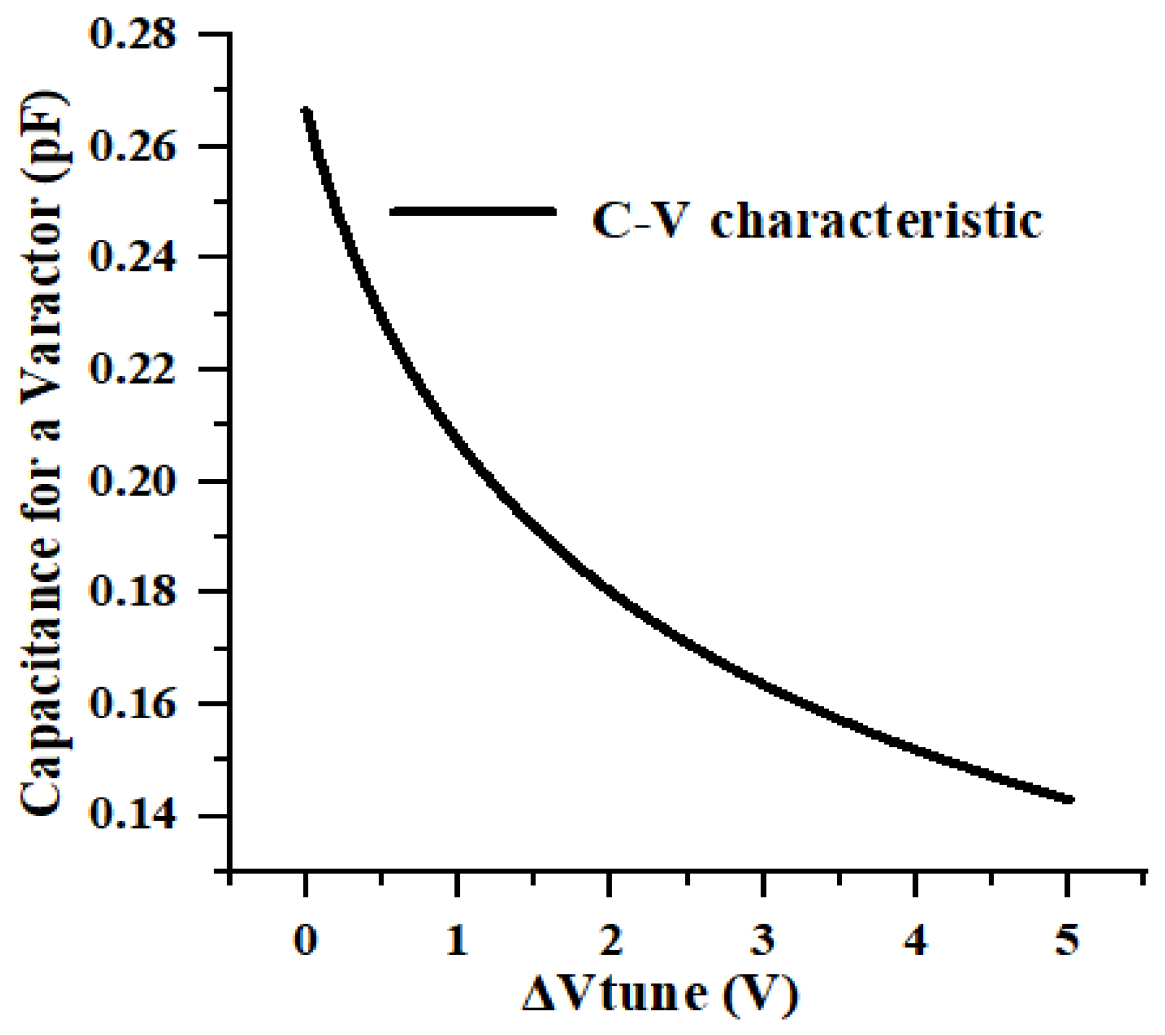
C-V characteristic of the varactor diode.

A balanced Colpitts VCO prototype was designed and fabricated to validate its performance. Figure 8 presents a micro-photograph of the fabricated VCO. Based on the measured oscillation frequency depicted in Figure 9, the proposed VCO can attain a wide tuning range of 33%, spanning from 2.56 GHz to 3.57 GHz. The measured frequency spectrum and phase noise for the fabricated VCO are presented in Figure 10 and Figure 11, respectively. Evidently, this VCO achieves an extremely low phase noise of −136.25 dBc/Hz at a 1 MHz offset from 2.95 GHz. The measured frequency-tuning characteristic shows that *K*_VCO_ remains nearly constant with small fluctuations across tuning voltages ranging from 0 V to 4 V. A marked increase in *K*_VCO_ can be observed when the tuning voltage rises to 5 V (VDD), which originates from the *C*–*V* behavior of the varactor diode. At a tuning voltage of 5 V, the voltage across the varactor approaches zero. At this bias point, the capacitance of the varactor changes rapidly with applied voltage, as reflected by its *C–V* characteristic in Figure 12.

To evaluate the sensitivity of the proposed VCO to temperature and supply voltage variations, simulations over a temperature range from −40 °C to +85 °C and supply voltages ranging from 0.9 × VDD to 1.1 × VDD (i.e., ±10% variation relative to the nominal VDD) were performed. According to the simulation results, increasing the supply voltage provides a moderate phase-noise improvement (~1 dB), though this gain comes at the cost of higher DC power consumption. As the temperature rises to +85 °C, phase noise deteriorates (~1.5 dB), mainly due to the elevated thermal noise of active devices. Overall, the VCO sustains robust oscillation across the full temperature range of −40 °C to +85 °C and under ±10% supply voltage variation.


(6) $$\mathrm{FOM}=\mathrm{PN}-20\log\left(\frac{f_{\mathrm{osc}}}{f_{\mathrm{offset}}}\right)+10\log\left(\frac{P_{\mathrm{DC}}}{1\mathrm{mW}}\right)$$

(7) $${\mathrm{FOM}}_{T}=\mathrm{PN}-20\log\left(\frac{f_{\mathrm{osc}}}{f_{\mathrm{offset}}}\right)+10\log\left(\frac{P_{\mathrm{DC}}}{1\mathrm{mW}}\right)-20\log\left(\frac{\mathrm{FTR}}{10}\right)$$

Table 3 summarizes some typical characteristics (figure of merit (FOM)/figure of merit including tuning range (FOMT), frequency-tuning range, DC power consumption (PDC), and phase noise) of the proposed VCO and several VCOs previously published in recent years. Evidently, the proposed VCO exhibits excellent performance characteristics, such as low phase noise, a wide tuning range, and satisfactory FOM/FOMT values. Generally, the VCOs based on Si-bulk technologies (CMOS/BiCMOS) in references [1,2,3] have advantages in terms of power consumption and tuning range, but their phase-noise performance is less than satisfactory. In contrast, the VCOs utilizing GaAs HBT or Bi-HEMT technologies in references [4,5,6] can achieve superior phase noise, yet they suffer from high power consumption, which leads to poor FOM and FOMT values. Compared with our previous work [11], substantial enhancements were achieved across several dimensions. First, the chip area has been drastically reduced from 1.6 mm^2^ down to 0.72 mm^2^, achieving an around 55% area reduction, which is highly desirable for compact satellite payloads with strict size constraints. Second, the frequency-tuning range has extended from 21% to 33%, corresponding to a 57% relative increase in tuning bandwidth, while DC power consumption has been slightly reduced from 45 mW to 42 mW (≈7% power reduction), and the central oscillation frequency has increased further. Third, tuning linearity has improved, which effectively suppresses (*K*_VCO_) fluctuation and benefits the stability of satellite PLL frequency synthesizer systems. Fourth, the overall design time has been significantly shortened.

As shown in Table 3, the proposed VCO can achieve lower phase noise, and its FOM and FOMT values are comparable to those of VCOs based on Si-bulk technologies. Additionally, when compared with VCOs implemented using GaAs technologies (HBT and BiHEMT), the FOM and FOMT of the proposed VCO are significantly better.

## 4. Conclusions

A balanced Colpitts VCO implemented using the GaAs BiHEMT process is presented and experimentally verified in this work. The noise-shifting technique and a three-bit switch were employed to enhance the phase-noise performance and expand the tuning range, respectively. To facilitate the design process and further enhance performance, a deep machine learning optimization algorithm was employed in the inductor design. The measurement results indicate that this VCO can be effectively integrated into a frequency synthesizer in satellite communication systems owing to its outstanding characteristics, including a wide tuning range from 2.56 GHz to 3.57 GHz and a low phase noise of −136.25 dBc/Hz at a 1 MHz offset from 2.95 GHz.

## Figures and Tables

**Table 1:** Prediction accuracy of the DNN surrogate model on the held-out test set.

Output	MAE	Relative MAE	*R* ^2^
L	33.2 pH	5.31%	0.978
Q	0.67	4.73%	0.930
SRF	0.407 GHz	2.31%	0.909

**Table 2:** Relative prediction errors of the DNN surrogate model in the interior, edge, and corner regions of the geometrical design space.

Region	L Relative MAE	Q Relative MAE	SRF Relative MAE
Interior	5.49%	4.14%	1.96%
Edge	5.24%	5.41%	2.04%
Corner	4.97%	5.38%	2.96%

**Table 3:** VCO performance comparison.

Ref	Tech.	f_osc_	FTR	P_DC_	PN	FOM	FOM_T_
[1]	CMOS	2.49	65.4	15.6	−128.5	−184.5	−200.8
[2]	BiCMOS	2.48	28.4	2.65	−124.8	−188.5	−197.6
[3]	CMOS	2.25	11	3.23	−119.4	−181.3	−182.1
[4]	GaAs HBT	3.74	23	300	−135.4	−180.6	−187.9
[5]	GaAs BiHEMT	2.4	17.9	27	−122	−175.3	−180.4
[6]	GaAs HBT	1.63	13.8	75	−134.6	−180.5	−183.3
This Work	GaAs BiHEMT	2.95	33	42	−136.3	−189.4	−199.8

*f_osc_* (GHz): oscillation frequency; *FTR* (%): frequency-tuning range; *P*_DC_ (mW): power consumption; *PN* (dBc/Hz@1 MHz): phase noise.

## Data Availability

The original contributions presented in the study are included in the article. Further inquiries can be directed to the corresponding authors.

## References

[1] Zou W., Zou X., Ren D., Zhang K., Liu D., Ren Z. (2019). 2.49–4.91 GHz wideband VCO with optimised 8-shaped inductor. Electron. Lett..

[2] Jang S.-L., Wang J.-J. (2017). Low-phase noise Class-C VCO with dy-namic body bias. Electron. Lett..

[3] Wang X., Yang X., Xu X., Yoshimasu T. 2.4-GHz-Band Low-Voltage LC-VCO IC with Simplified Noise Filtering in 180-nm CMOS. Proceedings of the 2016 IEEE MTT-S International Wireless Symposium (IWS).

[4] Florian C., Angelo S.D., Resca D., Scappaviva F. A Chip Set of Low Phase Noise MMIC VCOs at C, X and Ku band in InGaP-GaAs HBT Technology for satellite telecommunications. Proceedings of the IEEE MTT-S International Microwave Symposiym (IMS).

[5] Lu C.-H., Lin C.-H., Liao Y.-H., Chang H.-Y., Wang Y.-C. Monolithic 2-um/0.5-um GaAs HBT-HEMT (BiHEMT) Process for Low Phase Noise Voltage Controlled Oscillators (VCOs). Proceedings of the 2012 Asia Pacific Microwave Conference Proceedings.

[6] Shrestha B., Kim N.-Y. Double Cross Coupled Colpitts VCO with Low Phase Noise using InGaP/GaAs HBT Technology. Proceedings of the IEEE Radio Frequency Integrated Circuits Symposium.

[7] Liu H., Zhu X., Boon C.C., Yi X., Mao M., Yang W. (2014). Design of Ultra-Low Phase Noise and High Power Integrated Oscillator in 0.25-µm GaN-on-SiC HEMT Technology. IEEE Microw. Wirel. Compon. Lett..

[8] Sad C., Michailidis A., Noulis T., Siozios K. (2023). A Hybrid GA/ML-Based End-to-End Automated Methodology for Design Acceleration of Wireless Communications CMOS LNAs. Electronics.

[9] Karahan E.A., Liu Z., Sengupta K. (2023). Deep-Learning-Based Inverse-Designed Millimeter-Wave Passives and Power Amplifiers. IEEE J. Solid-State Circuits.

[10] Cao W., Gao J., Ma T., Ma R., Benosman M., Zhang X. (2025). Robust and Efficient Analog Circuit Parameter Optimization With Knowledge-Infused Reinforcement Learning. IEEE Trans. Comput.-Aided Des. Integr. Circuits Syst..

[11] Chau Q.T., Chen T., Huang W., Schwitter B.K., Gomez-Garcia R., Zhu X. (2026). AI-Assisted Automated Design of Multi-Stage MMIC Power Amplifiers in 150-nm GaN-on-SiC Technology. IEEE Trans. Circuits Syst. I Regul. Pap..

[12] Karahan E.A., Liu Z., Gupta A., Shao Z., Zhou J., Khankhoje U., Sengupta K. (2024). Deep-learning enabled generalized inverse design of multi-port radio-frequency and sub-terahertz passives and integrated circuits. Nat. Commun..

[13] Xia X., Cheng X., Chen F., Luo X., Deng X. (2020). An Improved Colpitts VCO with Low Phase Noise Using a GaAs BiHEMT Process. IEEE Microw. Wirel. Compon. Lett..

[14] Lee T.H. (2004). The Design of CMOS Radio-Frequency Integrated Circuits.

